# Awareness and Uptake of COVID-19 Vaccines Among the Residents of Bisha in Saudi Arabia

**DOI:** 10.7759/cureus.66265

**Published:** 2024-08-06

**Authors:** Mohammed Nawaf Alghamdi, Mohammed Abdullah Alharthi, Alyazeed Abdulrahman Alsamhoud, Bandar Khader Khabti, Mohammed Thamer Alshahrani, Sultan Saber Alharethi, Mohammed Mubarek Alshamrani, Obaid Faham Alharthi, Abdullah Hassan Alhalafi

**Affiliations:** 1 College of Medicine, University of Bisha, Bisha, SAU; 2 Department of Family and Community Medicine, University of Bisha, Bisha, SAU

**Keywords:** awareness, bisha, campaigns, covid-19, vaccination

## Abstract

Objective: Even though COVID-19 is no longer in an acute pandemic phase, sustaining awareness and promoting the uptake of COVID-19 vaccines are crucial for preventing future outbreaks and protecting public health. This study explores the awareness and uptake of COVID-19 vaccines among residents of Bisha, Asir province. It examines sources of information, healthcare providers' influence, and community engagement initiatives. The findings can inform future public health policies and strategies, supporting efforts to control the spread of the pandemic and enhance community resilience.

Methods: The cross-sectional study was administered to the adult population in Bisha. The study involved a total of 220 respondents. Data were collected using a questionnaire and analyzed using SPSS version 27 to obtain essential insights.

Results: Most respondents (n = 204, 92.73%) reported receiving information about COVID-19 vaccines from healthcare professionals, other people, and family members. The study also revealed that only 46 (20.9%) individuals had exposure to COVID-19, with 36 (78.26%) reporting infection before vaccination and only 10 (21.74%) reporting infection after vaccination. Regarding COVID-19 vaccine first-dose uptake, less than half (27.73%) of the respondents had received the first dose. Among those who took the first dose, 29 (47.54%) took the second dose, while 32 (52.46%) did not. It also shows a statistically significant difference in COVID-19 vaccine uptake based on the participant's age, gender, education level, comorbidity, smoking status, and place of residence (P < 0.05).

Conclusion: There was a significantly high level of awareness about the vaccine, with significant proportions of respondents indicating their willingness to take preventive measures even after vaccination. However, vaccine uptake remains below optimal levels, with various reasons cited for non-vaccination, including concerns about vaccine availability, efficacy, and side effects. Healthcare professionals should intensify public education efforts and ensure the availability of vaccines in various forms at hospitals to address this issue.

## Introduction

Since its emergence in late 2019, the severe acute respiratory syndrome coronavirus 2 (SARS-CoV-2) has caused the COVID-19 pandemic, leading to unprecedented global health challenges [1]. The virus spread quickly, prompting global efforts to develop effective vaccines [2]. Several vaccines received emergency use approval within the first year, marking significant milestones. To date, these vaccines have been pivotal in reducing the incidence of severe illness, hospitalizations, and deaths associated with the virus [3].

Like other countries, the Kingdom of Saudi Arabia faced significant social and economic challenges during the pandemic [4]. The overwhelming demand for health services and professionals puts immense pressure on public health. To curb the spread of the virus, Saudi Arabia implemented stringent measures like lockdowns, social distances, and movement restrictions [5]. As effective vaccines became available, the Saudi government, in collaboration with the Ministry of Health (MOH), initiated extensive vaccination campaigns to achieve widespread nationwide immunization [5]. Healthcare professionals used public venues, such as radio and television, to launch awareness campaigns promoting mass vacations in Saudi Arabia. Social media, as well as community outreach programs, were also used to increase awareness. The advertisements informed people about the efficacy of COVID-19 vaccinations to increase public acceptance of the vaccine. They also dispelled the myths and misinformation leading to vaccination hesitance [6]. Vaccine hesitancy has always been a barrier to immunization, not just for COVID-19 but also for other illnesses. Cultural norms, misinformation, and the perceived risk of vaccination influence some barriers, as defined by the World Health Organization (WHO) [7].

In Saudi Arabia, the commonly administered COVID-19 vaccines included the Pfizer-BioNTech, Moderna, and AstraZeneca-Oxford vaccines [8]. Preliminary research has revealed varying acceptance levels across different geographical areas and demographic groups among the Saudi Arabian population [4,8]. Some international studies have highlighted the importance of effective communication strategies and public trust in health authorities as a means of raising public awareness of the COVID-19 vaccine uptake [9,10].

The Bisha area in Asir province exhibits a distinctive population composition, including both urban and rural inhabitants, along with distinct socioeconomic demographic features. Before implementing awareness campaigns in any region, it is imperative to understand the residents' awareness and attitudes toward the COVID-19 vaccine. This includes examining the sources of information that shape public perceptions, the role of healthcare providers in influencing vaccine decisions, and the impact of community engagement initiatives. This study seeks to provide a detailed exploration of the awareness and uptake of the COVID-19 vaccine among residents of Bisha, offering insights that can inform future public health policies and strategies in the region. Through this research, we aim to support the ongoing efforts to control the pandemic and enhance the resilience of communities against current and future public health threats.

## Materials and methods

Study design

This study utilized a cross-sectional design between December 2023 and May 2024, encompassing the adult population of Bisha, Saudi Arabia. Its primary objective was to assess the awareness of and uptake of the COVID-19 vaccine among Bisha residents.

Inclusion and exclusion criteria

The inclusion criteria for this study were individuals living in the Bisha region who agreed to participate and were 18 or older. On the other hand, the exclusion criteria included individuals residing outside the Bisha Region, those who refused to participate, and those younger than 18 years old.

Sampling technique

The study employed a non-probability convenience sampling technique, selecting respondents based on their availability and willingness to participate during the study period.

Sample size

The Cochrane sample size formula was employed to ascertain the sample size. The formula follows:

n = Z2(1 − p)/d2,

where n represents the sample size, Z denotes the critical for 95% CI, 85% reflects the prevalence of acceptance, and d indicates the margin of error (5%). Initially, the minimum required sample size was 196. However, to enhance reliability, we opted to work with a slightly larger sample size of 220 respondents.

Data collection and procedures

The data were collected using a self-administered survey connected to the database through social media. The data for this study was sourced only from residents in the Bisha region. The data were categorized into three distinct groups: (1) sociodemographic variables, including age, gender, socioeconomic status, and educational achievement; (2) awareness and willingness to receive the booster dose; and (3) the reliability of health information obtained from the Internet. A digital survey was built using Google Forms (Google LLC, Mountain View, California, United States) and then distributed via various social media channels, with a particular emphasis on the Bisha region. These sites included Twitter, WhatsApp, and Telegram. The spreadsheet facilitated the generation of responses, while the surveys ensured the respondents’ names were kept confidential. The hyperlink remained operational for twenty days, during which the collected responses were integrated into the study after eliminating any missing entries. Redundant records associated with identical email addresses were omitted. A pre-tested survey by Ahmad and colleagues was used for this study [11].

Data analysis

After completing the data collection process, we input the gathered data into Excel spreadsheets (Microsoft Corporation, USA) to eliminate missing entries, duplicates, and outliers. The data were coded and transferred to IBM SPSS Statistics for Windows, Version 27.0 (released 2020, IBM Corp., Armonk, NY) for analysis. Categorical variables were summarized using counts and frequencies, while means and standard deviations were utilized for continuous variables. Associations between categorical variables were evaluated using the Chi-square test, with statistical significance determined at a threshold of p < 0.05.

Ethical considerations

The Bisha University Ethics Committee granted the ethical clearance under the reference of BU/1814/223 (22/03/2021). To maintain anonymity, personal identifiers were omitted from the collected data, ensuring no connection between individual identities and study outcomes. Rigorous confidentiality protocols were implemented, and the participants provided informed consent willingly, without coercion, after being briefed on the study's objectives, procedures, associated risks, and potential benefits.

## Results

Table 1 displays the demographic attributes of the 220 people involved in the investigation. Among the entire group of participants, 103 (46.8%) were between the ages of 18 and 29. The sample exhibited a mostly female composition, with 145 (65.91%) individuals identifying as women and 75 (34.09%) individuals identifying as men. The group exhibited a diverse array of educational attainment, with the highest proportion of participants, 107 (48.64%) individuals, having successfully finished high school. Subsequently, 74 (33.64%) individuals had completed further levels of education. Of the total, just 39 (17.72%) acquired a university degree. Regarding their income position, a majority of the participants (123 or 55.9%) stated that they earn less than 5000 riyals. Moreover, most participants, precisely 198 (90.0%), had no additional medical conditions. Furthermore, out of the total number of participants, 178 (80.9%) did not smoke, 207 (94.1%) were Saudi Arabian citizens, and 118 (53.6%) lived in rural areas. 

**Table 1:** Demographic characteristics of the participants (n = 220) Data are presented as n and %.

Characteristics	Category	n (%)
Age	18-29	103 (46.8%)
	30-49	87 (39.5%)
	50-69	22 (10%)
	Above 70 years	8 (3.7%)
Gender	Male	75 (34.09%)
	Female	145 (65.91%)
Education	High school	107 (48.64%)
	University	39 (17.72%)
	Other	74 (33.64%)
Income status	<5000 Riyal	123 (55.9%)
	5000-10000 Riyal	75 (34.1%)
	>10000	22 (10%)
Comorbidity	Yes	22 (10%)
	No	198 (90.0%)
Smoking	Yes	24 (10.9%)
	No	178 (80.9%)
	I quitted	18 (8.2%)
Nationality	Saudi	207 (94.1%)
	Non-Saudi	13 (5.9%)
Place of residence	City	102 (46.4%)
	Villages	118 (53.6%)

Table 2 reveals that a significant majority of respondents, namely, 204 individuals (92.73%), said that they acquired knowledge about COVID-19 vaccines from healthcare professionals, as well as from other individuals and family members. Moreover, a significant proportion of respondents (184 or 83.6%) knew that the vaccines did not include a viable coronavirus variant. Many individuals reported that naturally acquired immunity is way better than immunity obtained through the COVID-19 vaccines. In addition, a significant majority of 161 (73.2%) individuals recognized the ongoing importance of adhering to preventive measures, such as wearing masks and maintaining social distance, even after receiving the COVID-19 vaccine. Moreover, most participants, specifically 176 individuals (80.0%), believed that immunizations were indispensable for all individuals, irrespective of age or individuals with weakened immune systems. Furthermore, most (158, 71.8%) participants concurred that individuals who had already contracted COVID-19 should receive the vaccine.

**Table 2:** Awareness of COVID-19 vaccine among the respondents (n = 220) Data are presented as n and %.

Research item	Category	n (%)
Has anyone informed you about the COVID-19 vaccine?	Yes	204 (92.73%)
	No	16 (7.27%)
If someone has informed you about the vaccine, did they advise you to take the vaccine?	Yes	149 (73.04%)
	No	55 (26.96%)
If someone has informed you, who was the person?	Healthcare professional	94 (63.1%)
	Family member	22 (14.8%)
	other	33 (22.1%)
Do you believe that a live form of the coronavirus is included in the vaccines?	Yes	10 (45.5%)
	No	184 (83.6%)
	I don’t know	26 (11.8%)
Do you believe that immunity developed naturally is preferable to immunity acquired using the Covid-19 vaccine?	Yes	16 (7.3%)
	Probably	25 (11.4%)
	No	149 (67.7%)
	I don’t know	30 (13.6%)
Do you really believe that receiving a COVID-19 vaccination will allow you to forget about wearing a mask and social distancing?	Required to continue taking precautions	161 (73.2%)
	After taking vaccines, it is no longer needed	7 (3.2%)
	I don’t know	52 (23.6%)
Are vaccines only required for the elderly and those with weakened immune systems, in your opinion?	Yes	7 (3.2%)
	No	176 (80.0%)
	I don’t know	37 (16.8%)
Do you believe that someone who has already had COVID-19 doesn't need to get vaccinated?	They also need to take	158 (71.8%)
	There is no need	22 (10.0%)
	I don’t know	40 (18.2%)

Table 3 reveals that COVID-19 exposed 46 individuals, representing 20.9% of the population. Out of the total number of participants, 10 (21.74%) individuals (21.74%) contracted the infection after receiving the immunization, whereas 36 (78.26%) individuals (78.26%) contracted the infection before receiving the vaccine. Just 27.73% of the participants (61 individuals) received their initial dose of the COVID-19 vaccine, which is less than half of the total number of responses. Out of the total number of individuals who received the first dose, 29 (47.54%) proceeded to receive the second dose, whereas 32 (52.46%) did not. Pfizer was the most frequently used vaccine, with 22 participants (36.07%), followed by Oxford (16 participants (26.23%), Moderna (14 participants (22.95%), and Janssen (nine participants (14.75%). Most individuals in Bisha (58, 95.08%) received the vaccine. The primary justifications for vaccination were personal decision (17.87%), medical recommendation (16.23%), suggestions from others (11.03%), and other reasons (17.87%). The reasons for not obtaining the vaccine differed among individuals. Among all respondents, 66 individuals (41.51%) indicated a desire to receive the vaccine but were unable to do so. A total of 29 (18.24%) participants expressed doubts regarding the effectiveness of the vaccination. In addition, 23 (14.7%) individuals raised concerns about possible adverse effects. Fourteen (8.81%) individuals refrained from receiving the vaccine due to a lack of information. In addition, 12 (7.55%) individuals expressed a phobia of needles, nine (5.66%) individuals cited miscellaneous reasons, and six (3.77%) individuals expressed apprehension about contracting COVID-19 from the vaccine.

**Table 3:** Uptake of COVID-19 vaccine among the respondents (n = 220) Data are presented as n and %.

Characteristics	Category	n (%)
Have you had COVID-19 infection?	Yes	46 (20.9%)
	No	174 (79.1%)
If yes, when did you have COVID-19 infection?	Before vaccine	36 (78.26%)
	After vaccine	10 (21.74%)
Have you taken COVID-19 vaccine first dose?	Yes	61 (27.73%)
	No	159 (72.27%)
Second dose	Yes	29 (47.54%)
	No	32 (52.46%)
If yes, which vaccine:	Pfizer vaccine	22 (36.07%)
	oxford vaccine	16 (26.23%)
	Moderna vaccine	14 (22.95%)
	Janssen vaccine	9 (14.75%)
	none	0 (0.00%)
If yes, where did you take the vaccine?	In Bisha	58 (95.08%)
	Outside Bisha	3 (4.92%)
If yes, what was the reason?	Took on your own	17 (27.87%)
	Someone suggested	11 (18.03%)
	Doctor advised	16 (26.23%)
	Other	17 (27.87%)
If no, why not?	Want to take but vaccine not available	66 (41.51%)
	Scared of side effects	23 (14.47%)
	Scared of injections	12 (7.55%)
	Scared of getting COVID from the vaccine	6(3.77%)
	Don't think the vaccine is effective	29(18.24%)
	Don't have enough information about the vaccine	14(8.81%)
	Other reason:	9(5.66%)

Table 4 demonstrates a notable disparity in COVID-19 awareness across participants, depending on their degree of education and place of residence (p < 0.05).

**Table 4:** Factors associated with awareness of COVID-19 vaccine (n = 220) Data are presented as n and %. The chi-square test was used to test the significance. The p-value is considered statistically significant at p-value < 0.05.

Characteristics	Category	Yes n (%)	No n (%)	P-value*
Age	18-29	95 (46.6%)	8 (50.0%)	0.072
	30-49	83 (40.7%)	4 (25.0%)	
	50-69	19 (9.3%)	3 (18.8%)	
	Above 70 years	7 (3.4%)	1 (6.2%)	
Gender	Male	70 (34.3%)	5 (31.3%)	0.161
	Female	134 (65.7%)	11 (68.7%)	
Education	High school	99 (48.5%)	8 (50.0%)	0.015
	University	37 (18.2%)	2 (12.5%)	
	None	68 (33.3%)	6 (37.5%)	
Income Status	<5000	113 (55.4%)	10 (62.0%)	0.134
	5000-10000	69 (33.8%)	6 (37.5%)	
	>10000	20 (9.8%)	2 (12.5%)	
Comorbidity	Yes	18 (8.8%)	4 (25.0%)	0.260
	No	186 (91.2%)	12 (75.0%)	
Smoking	Yes	22 (10.7%)	2 (12.5%)	0.081
	No	167 (81.9%)	11 (68.7%)	
	I quitted	15 (7.4%)	3 (18.8%)	
Nationality	Saudi	194 (95.1%)	13 (81.2%)	0.213
	Non-Saudi	10 (4.9%)	3 (18.8%)	
Place of residence	City	99 (48.5%)	3 (18.8%)	0.018
	Villages	105 (51.5%)	13 (81.2%)	

Table 5 displays a significant disparity in accepting COVID-19 vaccines based on age, gender, education level, comorbidities, smoking status, and residence (p < 0.05).

**Table 5:** Factors associated with the uptake of COVID-19 vaccine (n = 220) Data are presented as n and %. The chi-square test was used to test the significance. The p-value is considered statistically significant at p-value < 0.05.

Characteristics	Category	Yes n (%)	No n (%)	P-value*
Age	18-29	24 (39.3%)	79 (49.7%)	0.003
	30-49	21 (34.4%)	66 (41.5%)	
	50-69	11 (18.1%)	11 (6.9%)	
	Above 70 years	5 (8.2%)	3 (1.9%)	
Gender	Male	19 (30.1%)	56 (35.2%)	0.001
	Female	42 (68.9%)	103 (64.8%)	
Education	High school	30 (49.2%)	77 (48.4%)	0.005
	University	24 (39.3%)	15 (9.4%)	
	None	7 (11.5%)	67 (42.2%)	
Income status	<5000	31 (50.8%)	92 (57.8%)	0.058
	5000-10000	24 (39.4%)	51 (32.1%)	
	>10000	6 (9.8%)	16 (10.1%)	
Comorbidity	Yes	13 (21.3%)	9 (5.7%)	0.004
	No	48 (78.7%)	150 (94.3%)	
Smoking	Yes	11 (18.1%)	13 (8.2%)	0.002
	No	44 (72.1%)	134 (84.3%)	
	I quitted	6 (9.2%)	12 (7.5%)	
Nationality	Saudi	56 (91.8%)	151 (95.0%)	0.063
	Non-Saudi	5 (8.2%)	8 (5.0%)	
Place of residence	City	32 (52.5%)	71 (44.0%)	0.013
	Villages	29 (47.5%)	89 (56.0%)	

## Discussion

The results of this study offer valuable insights into COVID-19 vaccine awareness and uptake among Bisha residents in Saudi Arabia. The high awareness rate of 92.73% is promising and suggests that public health initiatives, especially those involving healthcare professionals, have been largely effective. This finding aligns with other studies showing that healthcare workers are a crucial source of vaccine information and significantly impact vaccination decisions [12,13].

Most respondents understood that COVID-19 vaccines do not contain a live virus and recognized the importance of vaccination even after a prior infection. This awareness reflects successful educational campaigns but also highlights a persistent belief among some that natural immunity is superior to vaccine-induced immunity. Similar beliefs have been documented in other studies [14,15], indicating the need for ongoing education about the benefits of vaccination over natural immunity.

Despite high awareness, vaccine uptake in Bisha was relatively low, with only 27.73% receiving the first dose and even fewer completing the series. This is notably lower than uptake rates reported in recent studies by Asch and Nguyen et al. [16,17]. Asch et al. (2021) found that vaccine uptake in the United States was significantly higher, with many individuals receiving both doses promptly [18]. Nguyen et al. [19] also reported high vaccination rates in the United States, with factors such as accessibility and public health initiatives contributing to this success. Barriers such as vaccine availability and concerns about side effects were cited by those who were unvaccinated. These issues are consistent with findings from other regions, including Europe and the United States [20,21], underscoring the need for better vaccine distribution and targeted communication strategies.

The study also uncovers significant differences in vaccine awareness and uptake based on education level, place of residence, age, and gender. Higher education levels were associated with better vaccine awareness and uptake, supporting other research that links education with higher vaccine acceptance [22,23]. Urban residents showed higher vaccine awareness compared to those in rural areas, reflecting similar patterns observed globally where urban populations typically have better access to health information [24,25].

The differences in vaccine uptake among various age groups and genders, with younger people and males having lower uptake rates, align with existing literature. These groups often have varying perceptions of vaccine risk and efficacy [14,16]. Addressing these specific concerns through tailored public health messaging could help improve vaccination rates.

This study has several limitations that should be considered. Using a non-probability convenience sampling method means that the sample may not fully represent the broader population of Bisha, which could affect the generalizability of the results. In addition, the reliance on self-reported data for vaccine uptake and awareness introduces the possibility of response bias, as participants might inaccurately report their vaccination status or knowledge. The study also focused primarily on the most commonly administered vaccines in Bisha, potentially overlooking other vaccine types that could impact awareness and uptake. Moreover, the cross-sectional design provides only a view of vaccine attitudes and behaviors simultaneously, limiting the ability to track changes or trends over time. Lastly, the study may not have fully captured the influence of cultural and socioeconomic factors on vaccine perceptions and availability, which could differ across various regions of Saudi Arabia.

## Conclusions

The study offered an in-depth examination of COVID-19 vaccine awareness and uptake among residents of Bisha, Saudi Arabia. It revealed that most people were well-informed about the vaccine, mainly due to guidance from healthcare professionals, and understood its benefits and the importance of ongoing precautions. Despite this high level of awareness, vaccine uptake remained low, with fewer than 30% of participants receiving the first dose and even fewer completing the vaccination series. Factors like education, place of residence, age, and gender played significant roles in both awareness and uptake, with higher education and living in urban areas being linked to better vaccine adoption and awareness.
